# Distinct Tau Pathologies in the Nucleus Basalis of Meynert between Early-Onset and Late-Onset Alzheimer’s Disease Patients Revealed by Positron Emission Tomography

**DOI:** 10.1192/j.eurpsy.2025.1670

**Published:** 2025-08-26

**Authors:** H. Suzuki, K. Tagai, M. Ono, H. Shimizu, H. Endo, H. Matsumoto, Y. Kataoka, S. Moriguchi, S. Kurose, M. Ichihashi, H. Shinotoh, N. Kokubo, Y. Momota, T. Tokuda, M. Onaya, M. Mimura, N. Sahara, A. Kakita, M. Higuchi, K. Takahata

**Affiliations:** 1 Psychiatry, NHO Shimofusa Psychiatric medical center; 2 Department of Functional Brain Imaging, Institute for Quantum Medical Science, Quantum Life and Medical Science Directorate, National Institutes for Quantum Science and Technology, Advanced Neuroimaging Center, Institute for Quantum Medical Science, National Institutes for Quantum Science and Technology (QST), Chiba; 3 Department of Pathology, Brain Research Institute, Niigata University, Niigata; 4 Neuropsychiatry, Keio university school of medicine, Tokyo, Japan

## Abstract

**Introduction:**

Alzheimer’s disease (AD) is characterized by the abnormal accumulation of amyloid-β and tau proteins. Previous studies have demonstrated that early-onset AD (EOAD) has more rapid and significant tau accumulation compared to late-onset AD (LOAD). Particularly, postmortem analyses have shown greater tau accumulation in the nucleus basalis of Meynert (nbM) in EOAD. However, there is a lack of clinical studies that directly compare tau pathologies in EOAD and LOAD or explore their associations with clinical symptoms.

**Objectives:**

To evaluate the tau accumulation patterns in the nbM and other brain regions defined by Braak stages (I/II, III/IV, V/VI) in EOAD and LOAD using 18F-florzolotau PET imaging. Additionally, to analyze the relationship between tau accumulation in the nbM and cognitive function.

**Methods:**

The study included 38 amyloid-positive AD patients (15 EOAD, 23 LOAD) and 46 healthy controls (HCs). PET scans with 18F-florzolotau were performed, and the standardized uptake value ratios (SUVRs) of tau accumulation in the nbM and Braak stage regions were calculated using the cerebellum as the reference. Cognitive assessments were conducted using the Mini-Mental State Examination (MMSE) and other neuropsychological tests. Postmortem brain tissue from six AD patients and two HCs was histologically analyzed to validate PET findings.

**Results:**

EOAD patients showed significantly higher tau accumulation in the nbM than LOAD patients (p = 0.004). The SUVRs in Braak stage regions also tended to be higher in EOAD (I/II: p = 0.244, III/IV: p = 0.120, V/VI: p = 0.079). Correlation analysis revealed no significant relationship between nbM SUVR and Braak stage SUVRs in EOAD, whereas LOAD patients exhibited positive correlations in Braak stages I/II (r = 0.50, p = 0.014) and III/IV (r = 0.43, p = 0.043). In LOAD, nbM tau accumulation correlated negatively with MMSE scores (r = -0.55, p = 0.006). In EOAD, higher Braak stage tau was associated with a stronger negative trend in MMSE (III/IV: r = -0.37, p = 0.178; V/VI: r = -0.41, p = 0.126). Histopathological examination confirmed the presence of ghost tangles in advanced AD and intracellular tau in early-stage AD, supporting the PET imaging results.

**Conclusions:**

The study highlights distinct tau pathology differences in the nbM and their impact on cognitive function between EOAD and LOAD. The findings suggest that while nbM tau pathology in LOAD is linked to disease severity, EOAD is influenced more by cortical tau pathology. PET imaging of tau provides a promising approach for enhancing diagnostic and therapeutic strategies for Alzheimer’s disease.

**Disclosure of Interest:**

None Declared

